# Isolated Avulsion Fracture of the Lesser Trochanter in an Adolescent: A Case Report and Review of the Literature

**DOI:** 10.7759/cureus.36693

**Published:** 2023-03-26

**Authors:** Dimitrios Pallis, Konstantinos Tsivelekas, Margarita-Michaela Ampadiotaki, Petros Nikolakakos, Stamatios A Papadakis

**Affiliations:** 1 Second Department of Orthopaedics, KAT General Hospital of Attica, Athens, GRC

**Keywords:** isolated, adolescent, fracture, avulsion, lesser trochanter

## Abstract

Avulsion fractures of the lesser or greater trochanter or the iliac crest are uncommon injuries in adolescents. The anterior superior iliac spine, ischium, and anterior inferior iliac spine are the most frequently affected sites. We report a rare case of a 14-year-old boy who sustained an avulsion fracture of the lesser trochanter while playing soccer. No malignancy or associated metabolic bone disease was detected. Conservative treatment was suggested, consisting of a non-weight-bearing period and analgesics. Routine follow-up was performed at one, three, and six months after the injury. Radiographs were utilized to confirm fracture healing. Full recovery and return to a pre-injury functional level were observed at six months. Within this timeframe, a thorough literature review is performed.

## Introduction

Avulsion fractures are identified as the result of an excessive amount of the musculotendinous unit forces or a sudden and violent muscular contraction across an on-growth apophysis. Mostly occurring during demanding sports activities, avulsion fractures can come with severe long-term post-traumatic limitations, especially in young athletes [1]. Adolescent football players and gymnasts represent the most affected groups, where the impact is greater for males. The variations in pelvic anatomy regarding both the skeleton maturation and the biomechanical complexity until adulthood determine to a significant extent that the pelvis is the most usually affected site. Secondary ossification centers of the pelvis are the most vulnerable sites, including the ischial tuberosity, the anterior inferior ischial spine, and the anterior superior iliac spine [2].

Avulsion fractures of the lesser trochanter, greater trochanter, and iliac crest are less common. Isolated avulsion fractures of the lesser trochanter account for less than 1% of hip injuries in adolescents [2]. As the result of forceful muscle contraction of the iliopsoas, the open growth plate is at greater risk of disruption because the musculotendinous unit and the attached Sharpey’s fibers are less susceptible to injury compared to the cartilaginous growth plate. Milch et al. suggested the term “apophysiolysis” for such injuries, concerning their similarities with epiphyseal injuries. Abrupt eccentric contraction or distention of the iliopsoas, typically among those who accelerate or decelerate during sports activities, is the most representative mechanism of injury [3]. 

Regarding the relatively rare frequency of isolated lesser trochanter fractures, only a few case reports and case studies have assessed the treatment options and rehabilitation of such patients in the literature. Traditionally, non-operative treatment has been the mainstay of management, providing sufficient functional outcomes [2]. However, there is still controversy concerning the beneficial potential of surgical treatment in several cases, either as a primary or secondary option [2,4]. We hereby present the case of a conservatively treated adolescent football player who sustained an avulsion fracture of the lesser trochanter during soccer. Within this frame of reference, a thorough literature review of isolated lesser trochanter fractures is performed.

## Case presentation

**Figure 1:**
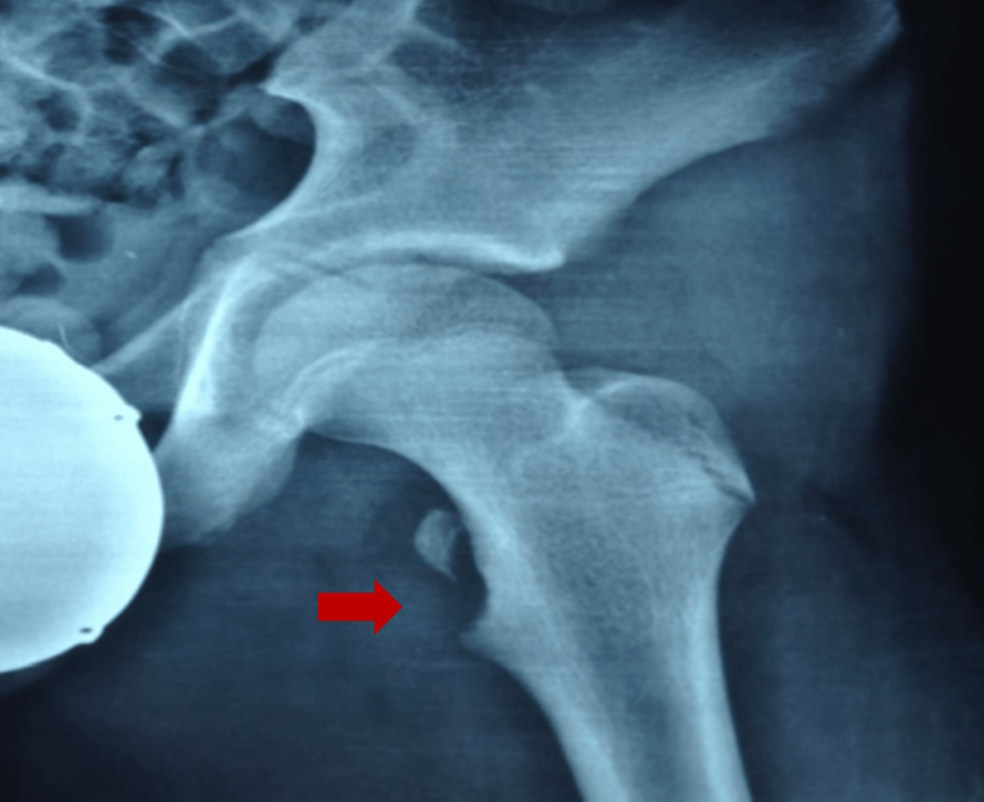
Anteroposterior radiograph of the left hip, showing the isolated avulsion fracture of the lesser trochanter (red arrow).

A 14-year-old boy presented to the pediatric orthopedic emergency department. Antalgic gait supported with crutches was detected upon presentation and attributed to acute-onset left groin pain after kicking the ball during football. Past medical history revealed no significant records. Acute groin pain after the injury forced him to interrupt the game and ask for assistance to leave the field as he was unable to bear weight on the left lower limb due to pain. While supine, the patient retained his leg in an antalgic position of flexion, adduction, and internal rotation. Returning the limb to the anatomic position was too painful, as was the extended leg rise maneuver. Clinical examination while sitting with both hips flexed at 90’ elicited pain with further flexion on the left side, giving a positive Ludloff test. Routine laboratory tests (FBC, ESR, CRP, liver, and renal function) were within normal ranges. The workup for the metabolic disease was negative. Radiological evaluation, including pelvic radiographs, revealed an avulsion fracture of the apophysis of the left lesser trochanter (Figure 1). Ultrasonography of the left hip detected the bone fragment with the attached tendon, and a CT scan of the left hip confirmed the isolated avulsion fracture of the lesser trochanter without additional pathologic findings. The displacement of the fracture was estimated at 1.5 cm.

**Figure 2:**
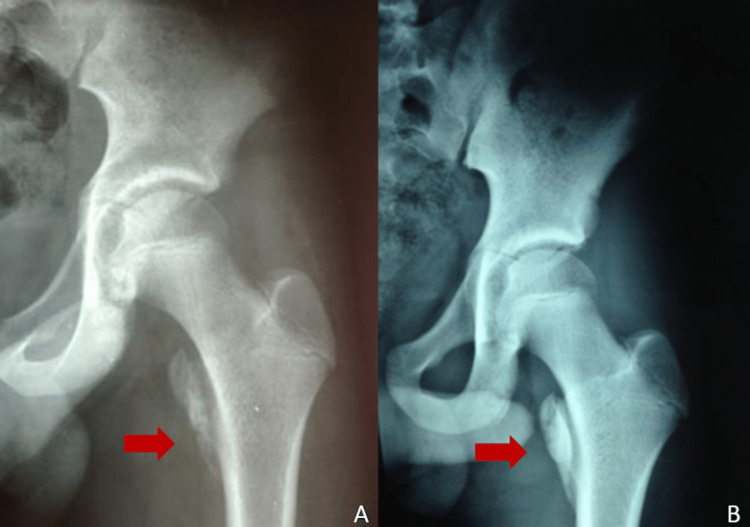
AP radiographs of the left hip at two (A) and six (B) months after the injury, indicating the callus formation in the posttraumatic period (red arrows).

Conservative treatment was recommended, consisting of non-weight bearing on the affected leg with crutches, aid, and analgesics. The patient was discharged after two days. Routine follow-up was performed in our outpatient clinic at four, eight, 12, and 24 weeks. Partial and gradually increased weight bearing was suggested at four weeks post-injury until reaching full weight bearing at six weeks. During the two-month follow-up, clinical symptoms had completely subsided, and the Harris Hip Score was 100 at three months post-injury (Figure 2A). The final reexamination at six months revealed that the patient had fully recovered and returned to the pre-injury activity level (Figure 2B).

## Discussion

Isolated fractures of the lesser trochanter represent less than 1% of hip injuries in adolescents and less than 3% of pelvic avulsion fractures. Hence, reports in the literature are scarce [2,5]. A definite traumatic event is almost always described by such patients, although minor trauma resulting in lesser trochanter avulsion fractures should raise the suspicion of underlying bone diseases, especially in adults. Further evaluation with MRI is warranted to rule out avulsions of non-ossified bone, undiagnosed malignancy, transient synovitis, and Legg-Calve-Perthes disease [6].

Treatment considerations concerning surgical or conservative treatment are mainly attributed to the lack of enough considerable evidence given the rare occurrence of such injuries [1,4,5]. Conventional management with non-operative treatment is traditionally the most frequent choice of treatment, providing sufficient outcomes in most cases. Volpi et al. published the largest series, with 30 conservatively treated patients suffering from isolated lesser trochanter fractures. The authors observed a complete return to the prior activity level at an average time of 11 weeks [2]. Similarly, Ruffing et al. mentioned successful rehabilitation in all conservatively treated patients in their series [7]. Metzmaker et al. described a five-stage, 60-day rehabilitation protocol following non-operatively treated patients with pelvic avulsion fractures. Almost all patients (88%) return to their pre-injury activity level, although only three of the studied patients presented with a lesser trochanter avulsion fracture [5]. Return to sports activities should be precisely considered since early return may result in re-injury and delayed recovery [2].

McKinney and Nelson proposed a classification system with regard to the relevant management. Conservative treatment is suggested for all types of acute lesser trochanter avulsion fractures (types 1-3). On the other hand, surgical intervention seems to be indicated in cases of exostosis or painful and symptomatic non-unions. Excision of exostosis followed by muscle reconstruction as well as large non-union fragments (>2cm) should be considered for treatment with internal fixation [8]. Eberbach et al. systematically reviewed the surgical versus conservative treatment of pelvic avulsion fractures. They concluded to a greater overall outcome in surgically treated patients with more than 1.5cm displacement of the fracture. However, only 1.8% of the 596 studied patients suffered from avulsion fractures of the lesser trochanter [9].

Though limited, literature reports describe the outcome of surgically treated avulsion fractures of the lesser trochanter [4,10]. Khemka et al. presented a great functional outcome following an arthroscopically assisted open reduction and internal fixation of avulsion fractures of the lesser trochanter. Fracture displacement greater than 2cm was detected in all patients, and cannulated screws were used to achieve reduction. Surgical considerations addressed by Khemka et al. were attributed to the potential development of non-union and strength decrease due to muscle shortening as the result of fracture displacement. Moreover, the authors mentioned ischiofemoral impingement (IFI) as a potential burden of conservative management of displaced avulsion fractures of the lesser trochanter [6]. Ischiofemoral impingement was first reported by Johnson in 1977. Johnson observed signs of IFI according to the decrease in distance between the lesser trochanter and the ischium due to fracture displacement [11]. To date, several operative procedures for internal fixation of the avulsion fractures of the lesser trochanter have been described, consisting of the suture anchor and cerclage fixation as well as cancellous screw fixation through open or arthroscopic approaches [4,10]. However, surgical intervention should be greatly reconsidered and guided by restricted indications since any surgical approach increases the risk of heterotopic ossification. Hence, such patients are in greater danger of potential IFI. Additionally, Homma et al. presented a conservatively treated case of a 14-year-old male with complete recovery to the prior level, regardless of the radiological detection of a non-union of the lesser trochanter [12]. Table 1 summarizes all literature reports of isolated avulsion fractures of the lesser trochanter.

**Table 1:** Review of lesser trochanter avulsion fractures in adolescents

Author (Year)	Number of Patients	Treatment	Outcome
Dimon (1972) [13]	30	Conservative	Satisfactory
Fasting et.al. (1978) [10]	1	Surgical	Good
Hösli et.al. (1995) [14]	3	Conservative	Good
Theologis et.al. (1997) [15]	3	Conservative	Good
Ruffing et.al. (2012) [16]	1	Conservative	Good
Papacostas et.al. (2013) [17]	1	Conservative	Good
Vazquez et.al. (2013) [18]	1	Conservative	Good
Goodbody et.al. (2014) [19]	36	Conservative	Satisfactory
Khemka et.al. (2014) [4]	3	Surgical	Good
Obi et.al. (2014) [20]	1	Conservative	Good
Homma et.al. (2015) [12]	1	Conservative	Good
McMillan et.al. (2016) [21]	1	Conservative	-
Grissa et.al (2017) [22]	1	Conservative	Good
Ruffing et.al. (2018) [7]	5	Conservative	Good
Tahir et.al (2019) [2]	3	Conservative	Good
Volpi et.al. (2021) [3]	30	Conservative	Good

## Conclusions

The conservative treatment of lesser trochanter avulsion fractures in adolescents seems to provide excellent results and a successful return to the previous level in less than 12 weeks. However, owing to the fact of the relatively low frequency of such injuries, current concepts are mostly structured on case reports and retrospective studies addressing conservative treatment as the optimal choice in most cases. Due to the scarcity of literature reports, a thorough comparison between surgical and conservative treatment methods is lacking. Hence, further prospective studies should be carried out, establishing the indispensable necessity for further research. 
